# Non-Destructive and Non-Invasive Measurement of Ethanol and Toxic Alcohol Strengths in Beverages and Spirits Using Portable Raman Spectroscopy

**DOI:** 10.3390/bios13010135

**Published:** 2023-01-13

**Authors:** Panagiota Papaspyridakou, Panagiota Giannoutsou, Malvina G. Orkoula

**Affiliations:** 1 Pharmaceutical Analysis Lab, Department of Pharmacy, University of Patras, 26504 Patras, Greece; 2 Institute of Chemical Engineering Sciences, Foundation for Research and Technology Hellas, 26504 Patras, Greece

**Keywords:** spirits, beverages, alcohol, toxicity, Raman, portable

## Abstract

The measurement of ethanol and toxic alcohol (methanol and isopropanol) strengths in beverages and spirits is crucial for health reasons but also for the identification of adulterated products. Many methodologies have been reported in the literature, based mainly on chromatographic and on spectroscopic techniques. Chromatographic techniques are laborious and time-consuming, while spectroscopic techniques are rapid and need no special sample pretreatment. All techniques were only applied to off-line or at-line manner. In the present work, Raman spectroscopy was used for fast and non-destructive measurements. A “through the container” method was developed for a non-invasive analysis, i.e., analysis without unsealing the bottles. This method, coupled with a miniature portable Raman, can serve for in-line measurements in a production line. The optimum laser focus for maximum spirit signal and minimum glass-wall signal was investigated. Calibration curves for the alcohols of interest were constructed and validated. The limits of detections were calculated and proved to be lower than the legitimate values. The influences of the liquor color and the bottle color, shape, and thickness were checked. Twenty-eight alcoholic products were studied. The concentrations found were compared against the nominal values (from the bottle labels).

## 1. Introduction

For decades, alcohol content (ethanol, methanol, and isopropanol) in beverages and spirits has been evaluated in an indirect way using density measurements after distillation [1,2]. This approach has been abandoned as it lacks the accuracy needed for this type of analysis. Space has been given to gas (GC) and liquid (HPLC) chromatography [3,4], which are undeniably accurate techniques. However, they are time-consuming and presume an off-line type of analysis (i.e., they involve removal of a sample from the bottle). In addition, ethanol and methanol cannot be measured simultaneously due to the usual huge difference in their concentrations. Thus, sample pre-treatment is needed.

The determination of strength in alcoholic beverages and spirits is imperative due to customs legislation as well as intoxication in cases of abuse. Adulteration by alcohols other than ethanol (methanol or isopropanol) is also an issue that needs to be dealt with, as their consumption can have serious health consequences, leading to blindness or even death. Swift and on-the-spot measurement without the destruction of packaging or sample contamination would help to avoid all the previously described problems. Furthermore, applications in the quality-control procedure in the production line would be of great value.

Apart from chromatography, spectroscopic techniques such as UV/Vis [5], MIR [6,7,8,9,10,11,12,13], NIR [14,15,16,17,18], and Raman spectroscopy [1,17,19,20,21,22,23,24,25] have also been reported in the literature for alcohol determination. Several devices (flow cells, immersion probes, and sensors) have been employed. No special sample preparation was needed, but a sample was withdrawn from the bottle. For NIR and MIR techniques, the presence of sugars appeared as an obstacle for analysis as it interfered with the signal from ethanol.

Performing ethanol analysis in alcoholic drinks or beverages in a non-destructive way, i.e., without opening the bottles, is a major challenge. In the present work, portable Raman spectroscopy is used to accurately measure the ethanol content (strength) as well as methanol and isopropanol content (toxicity) in alcoholic beverages (wine and beer) and spirits (vodka, whisky, and gin) directly from the bottle without opening the container and withdrawing a sample to analyze. 

## 2. Materials and Methods

### 2.1. Samples Analyzed

#### 2.1.1. Standard Solutions 

For the construction of the ethanol calibration curve, standard solutions of absolute ethanol (Fisher Chemical, Waltham, MA, USA, 99.99%) in water (Elix 3, Millipore, Burlington, MA, USA, 15 MΩ.cm) were prepared in concentrations of 0, 25, 50, 75, and 100% vol. For the toxic alcohols, quantities were added in spirit samples (Ballantine’s whiskey, Pernod Ricard, Paris, France). For pure methanol (Fisher Chemical, 99.99%), concentrations of 0.20, 0.40, 0.80, 1.00, and 1.50% vol were used, and for pure isopropanol, (Fisher Chemical, 99.98%) concentrations of 0.10, 0.20, 0.30, and 0.40% vol were used. Predefined amounts of the liquids were accurately pipetted and mixed in all cases.

#### 2.1.2. Drinks

Alcoholic drinks, beverages, and spirits in mini-bar packages (50 mL) and bottles (up to 700 mL) with different contents of alcohol were purchased from various countries around the world and used in this study. They are all quoted in Table 1 with their strength, color, and bottle characteristics (material, color, and volume) noted. 

### 2.2. Raman Instrumentation and Spectra Collection

A portable Raman spectrometer (i-Raman Plus, B & W Tek, Plainsboro Township, NJ, U.S.A.), equipped with a fiber-optic probe accessory, was used for the study. The set-up is shown in Picture 1. The handling of this system requires wearing safety glasses for eye protection. The incident radiation was a laser line at 785 nm with a power of 350 mW. Each Raman spectrum was the sum of four accumulations. The exposure time was 20 s for each accumulation. Five replicate measurements were performed for each sample (standard solution or drink), each one from different spot. A “dark” spectrum was collected before any sample recording (at the same conditions with the samples spectra, setting the laser off) to eliminate any interference.

The drinks were analyzed directly in their containers (circular or rectangular). The same containers were used as sample holders for the standard solutions. A backside metal coated cuvette of 5 mm path length (QX, Hellma GmbH & Co. KG, Müllheim, Germany) was used for collection of some spectra for comparison purposes.

### 2.3. Raman Spectra Processing

**Figure 1:**
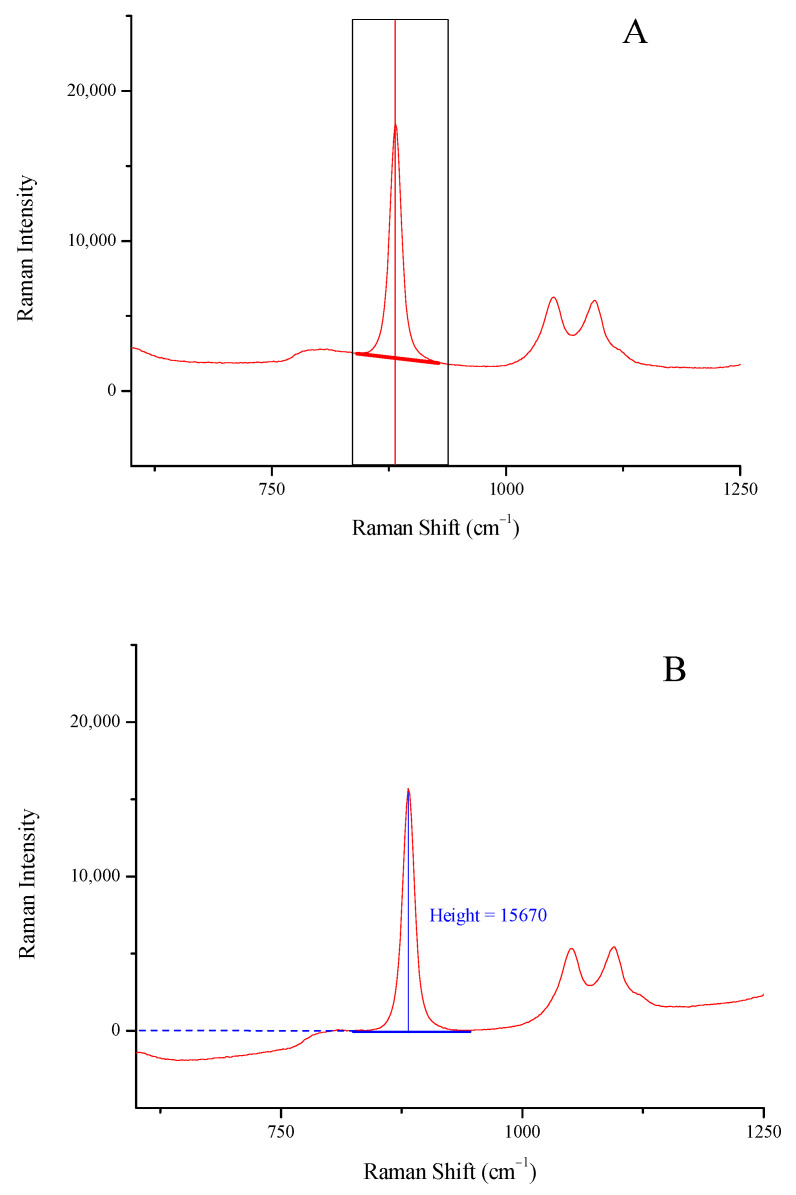
Drawing a two-point baseline (**A**), and background removal and height measuring (**B**) for the peak of ethanol at 878 cm^−1^.

The intensity (height) of the characteristic peaks of ethanol at 878 and 1046 cm^−1^, methanol at 1024 cm^−1^, and isopropanol at 814 cm^−1^ in all Raman spectra [20,24] were used after appropriate baselining and background correction (Figure 1). The peak boundaries used to draw the baseline were (approximately) 838–925 cm^−1^ for ethanol, 998–1147 cm^−1^ for methanol, and 796–829 cm^−1^ for isopropanol. 

### 2.4. Intraday and Interday Analyses

Intraday (three repeat cycles within the same day) and interday (three repeat cycles on the same day for three successive days) repeatability tests were performed for all standard ethanol–water, methanol–whiskey, and isopropanol–whiskey solutions. 

## 3. Results

### 3.1. Conventional Raman System

The usual way of analyzing a beverage or spirit with Raman spectroscopy is to use a special cuvette (Raman cuvette) made of synthetic quartz with a path length of 5 mm, equipped with a mirror in the back side, to reflect the scattered radiation and divert it towards the detector. A quantity of the sample to be tested is poured in, and the cuvette is placed under the Raman microscope in a vertical position or in a horizontal position in the case that an angle objective lens is used. This setup presupposes that the sample is withdrawn from the container and transported to the Raman lab for analysis.

### 3.2. Analysis of Raman Spectrum of a Spirit

**Figure 2:**
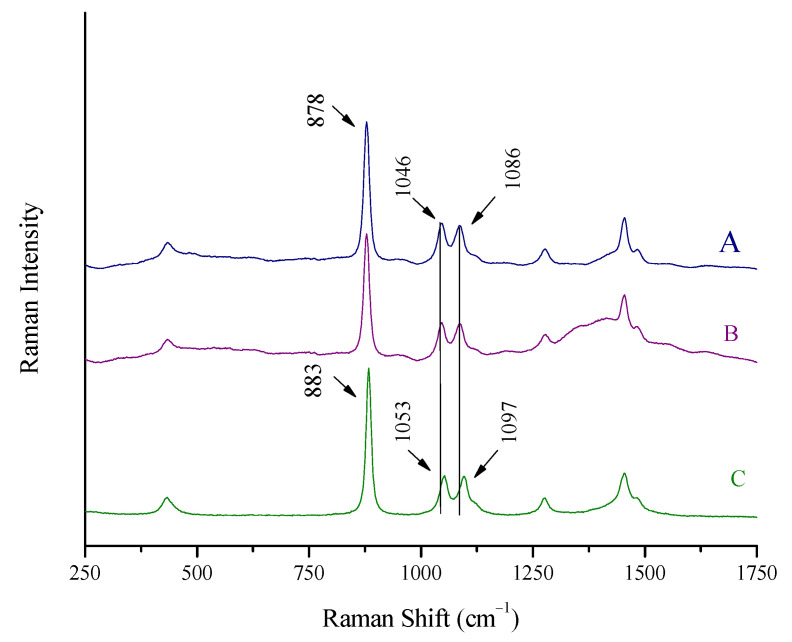
Raman spectra of (A) a spirit (whiskey, 43% vol) recorded using a Raman cuvette, (B) the same spirit using the Raman portable system and the “through the container” method, and (C) pure ethanol. Major ethanol peaks at 883, 1053, and 1097 cm^−1^ shifted to 878, 1046, and 1086 cm^−1^ for the whiskey.

The Raman spectrum of a spirit (whiskey), recorded as previously described, can be seen in Figure 2A. The Raman spectrum of pure ethanol, also recorded in a cuvette, is quoted in Figure 2C for comparison. The spectrum of the whiskey is dominated by the peaks of ethanol, which is its major constituent other than water (43% *v*/*v*). 

The peaks for pure ethanol, together with their assignment as reported in the literature [17,26,27], and the peaks of the whiskey are quoted in Table 2. 

It should be noted that a (red) shift of the maxima of the ethanol peaks at 883, 1053, and 1096 cm^−1^ to lower wavenumbers (878, 1046, and 1086 cm^−1^, respectively) is recorded for the whiskey spectrum. This is attributed to the strong hydrogen bonds between ethanol and water molecules in the spirit, which weaken the neighboring C-C, C-O, and C-H bonds and lead to polarizability changes [17,26,27]. 

### 3.3. Portable Raman Setup for Spectra Acquisition through Transparent Containers

**Figure 3:**
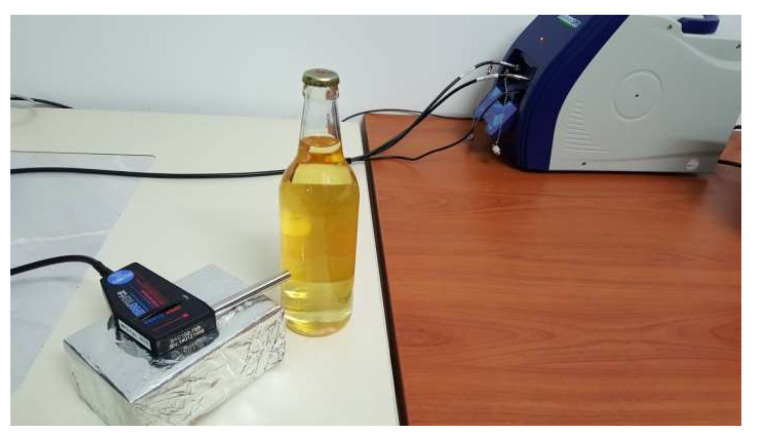
Portable Raman setup for the in-line analysis of drinks and spirits. A bottle of beer is placed as the sample.

In the present work, a different Raman setup was used to perform this type of analysis in a non-destructive and non-invasive way (Figure 3). The central unit, which houses the laser, the optics, and the detector, is a portable apparatus, small in volume and weight. The laser beam is directed to the sample through a fiber-optic probe. The scattered radiation is also collected from the probe and transferred through a second fiber-optic to the detector in the main unit. The spectra are pictured and processed on a laptop connected to the system using specialized software. (i-Raman Plus, B & W Tek, Plainsboro, NJ, USA). 

In this work, the portable Raman system was used for spectra acquisition from beverages and spirits through their container without package opening and sample withdrawal. It is important to note that a red laser (785 nm) was used to minimize the fluorescence of complex matrix spirits and beverages. Spectra were collected from different spots on the perimeter of the bottle. A characteristic spectrum of the whiskey (43% *v*/*v*) used before is shown in Figure 2B. Apart from a moderate background in the area of 1300–1500 cm^−1^, attributed to the glass wall, the quality of the spectrum is verified by its resemblance to the spectrum collected with a cuvette (Figure 2A). 

### 3.4. Optimization of the Signal

**Figure 4:**
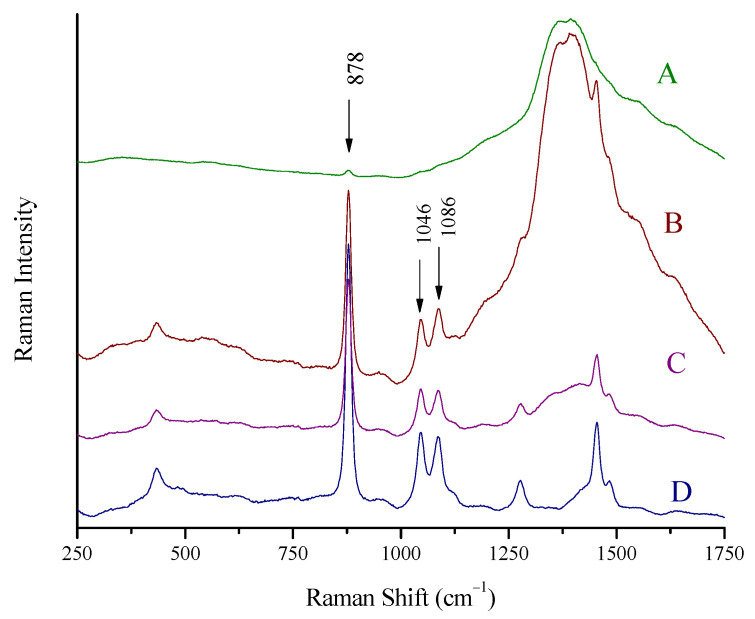
Raman spectra of a spirit (whiskey, 43% vol) recorded using the Raman portable system and the “through the container” method. (**A**) The laser is focused in front of the glass wall, (**B**) the laser is focused on the glass wall, (**C**) the laser is focused in the main volume of the liquid through the glass wall, and (**D**) Raman spectrum of the same spirit using the Raman cuvette. Major peaks of ethanol in the whiskey at 878, 1046, and 1086 cm^−1^ are marked.

The spectra of a spirit (whiskey, 43% vol) inside its container were recorded by placing the laser probe in three pre-specified distances away from the bottle so that the laser focused (A) on a point away from the bottle, (B) on the glass wall of the bottle, and (C) on a point inside the bottle. In all three cases, the probe was kept in a vertical position with respect to the glass wall, and the focal point moved from position (A) to (C) in a straight line. The spectra are shown in Figure 3. When the laser focused in front of the wall (position A), the wide peak of glass (1100–1750 cm^−1^) prevails in the collected spectrum (Figure 4A). The ethanol signal is suppressed to a minimum; the major peak at 878 cm^−1^ is barely seen, while the rest of the peaks have completely disappeared. In the case of laser focus exactly on the wall (position B), the spectrum collected is also dominated by the wide peak of glass. However, the peaks of ethanol (878, 1046, 1086 cm^−1^) can be clearly seen (Figure 4B). The optimum position is for the probe to be in contact with the glass wall so that the laser beam focuses inside the container, in the liquid. In this case, the signal from the glass is suppressed to a large extent and the signal from the spirit is clear (Figure 4C). The ethanol peaks are recorded almost as clear and intense as in the spectra from the cuvette (Figure 4D).

### 3.5. Raman Spectra of Drinks and Beverages

Various types of drinks were studied. The drinks, quoted in Table 1, varied with respect to their alcoholic strength, the type of bottle used (glass or plastic), and the thickness of the bottle. 

The previously described portable system was used for the collection of the spectra. The drinks were separated into two main categories: (A) colorless spirits in colorless transparent containers, glass or plastic and (B) colored spirits (color ranging from light yellow to dark red) in colorless glass containers.

#### 3.5.1. Colorless Spirits

**Figure 5:**
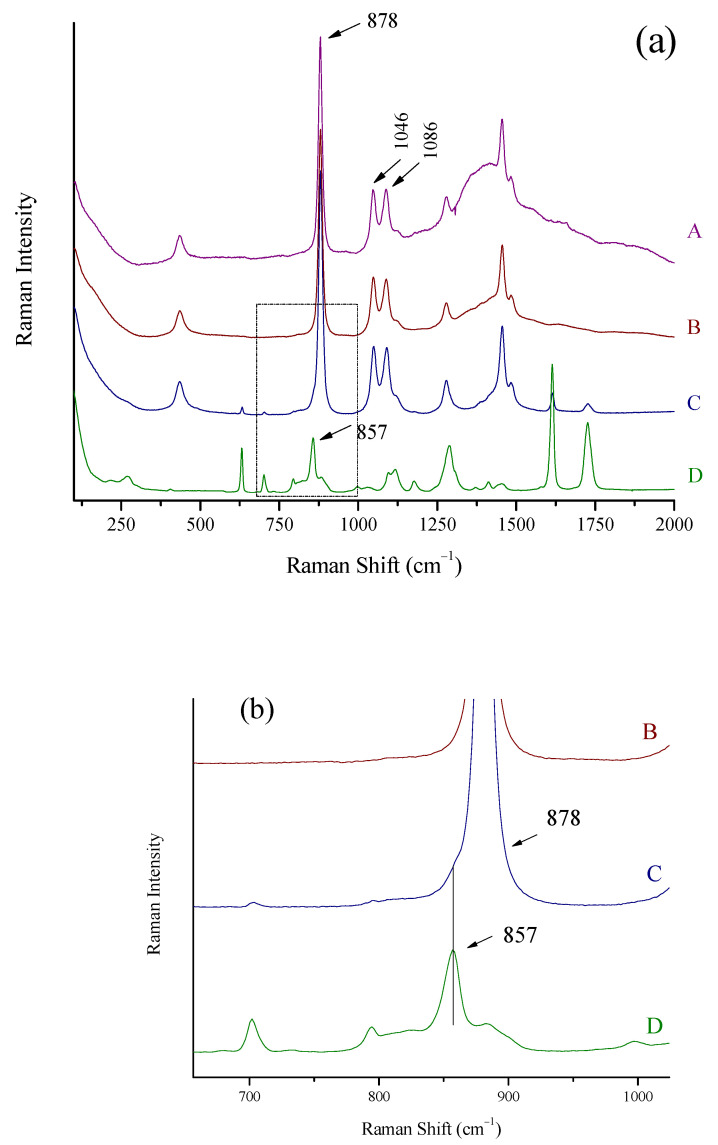
(**a**) Raman spectra of (A) ouzo, Varvagianni (42% vol), (B) vodka, Stolichnaya (40% vol), (C) gin, Beefeater (47% vol), and (D) the plastic container of Beefeater gin. The Raman portable system and the “through the container” method were used. Major ethanol peaks at 878, 1046, and 1086 cm^−1^ and the 857 cm^−1^ peak of the plastic bottle are marked. (**b**) Zoomed area of (**a**) in spectral area 700–1000 cm^−1^. The peak of plastic container at 857 cm^−1^ as a shoulder of the peak of ethanol at 878 cm^−1^ in the spectrum of Beefeater gin is shown.

Three representative strong spirits (ouzo, Varvagianni, 42% vol (A); vodka, Stolichnaya, 40% vol (B); and gin, Beefeater 47% vol, (C)) are shown in Figure 5a. All spectra were collected with the probe of the portable Raman system through their containers. Gin Beefeater had a plastic container, the spectrum of which is shown in Figure 5(aD). 

The ethanol peaks are sharp and clear for all the spirits. The background appearing in the spectra of ouzo, Vavagianni and vodka, Stolichnaya can, to a greater degree, be attributed to the thickness of the container which decreases from A to B (1.8 mm for A and 1.5 mm for B). This background does not, however, prohibit the detection of ethanol in the spirits.

For the Beefeater gin (Figure 5(aC)), the Raman signal of the plastic container is suppressed to minimum, in accordance to what was previously described for the glass with the “through the container” method. The peak of the plastic at 857 cm^−1^ appears only as a shoulder at the peak of ethanol at 878 cm^−1^ (Figure 5b).

#### 3.5.2. Colored Spirits

**Figure 6:**
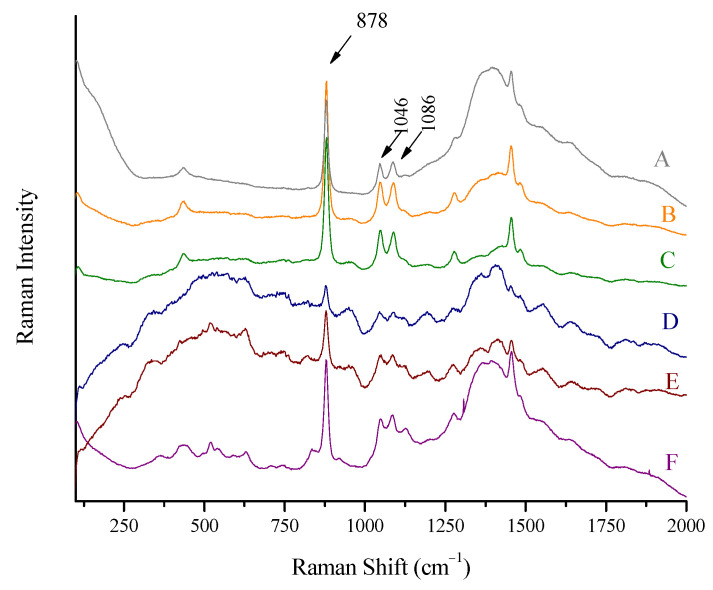
Raman spectra of (A) wine, Kourtaki (11.5% vol), (B) whiskey, Ballantine’s (43% vol), (C) whiskey, Jack Daniels (43% vol), (D) beer, Desperados (5.9% vol) (baselined), (E) liqueur, Fraise (baselined), and (F) liqueur, Belle Epoque, obtained using the Raman portable system and the “through the container” method. Major ethanol peaks at 878, 1046, and 1086 cm^−1^ are marked.

The Raman spectra of colored spirits and beverages are shown in Figure 6. The cases A to D refer to slightly colored (light yellow) but still transparent drinks in colorless, transparent glass bottles. Apart from the background at 1300–1500 cm^−1^, stemming from thicker glass walls, the ethanol presence is recorded. The spectrum of Desperados beer (D) is less intense as its ethanol content is low (5.9% vol). The peaks of the rest of the low-quantity ingredients can be seen. Some background in the area of 1250–1750 cm^−1^ also appears. 

The Belle Epoque liqueur is darker (orange) and not transparent. Its spectrum is also clear. The exact concentration is not known, but is expected to be around 40% vol. The intensity is not equal but is comparable to spirits of 43% vol concentration. On the other side, Fraise liqueur provided a spectrum of unexpectedly low intensity, taking into account the ethanol content (expected to be around 40% vol) when compared to Belle Epoque liqueur. This can be attributed to the deep red color of the spirit.

Spectra 5B and 5C can be compared directly, as they are two whiskeys of 43% strength. The container of B is thicker (1.6 mm) with a slightly higher glass peak, and C is thinner (1.4 mm) with slightly lower glass peak.

The background (spectral area 1250–1750 cm^−1^) is at a maximum in the spectra A and F of beverages in glass containers of 1.9 mm thickness and at a minimum in case C, with 1.4 mm glass thickness.

Spectra of other spirits of Table 1 are quoted in the Supplementary Materials.

### 3.6. Measuring Ethanol Strength 

#### Colorless Spirits

**Figure 7:**
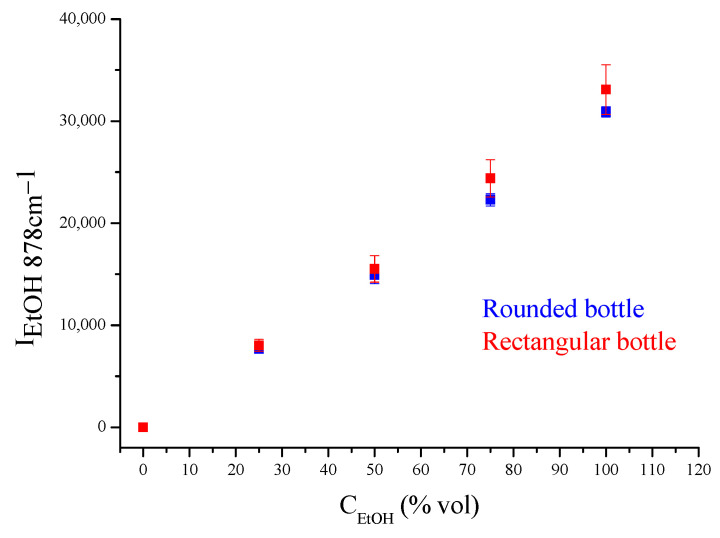
Intensity of the ethanol peak for ethanol–water standard solutions using a rectangular and a rounded bottle as sample holders.

For the quantification of ethanol in colorless spirits (Figure 5), a calibration curve was constructed. Standard solutions of absolute ethanol in water in concentrations of 0, 25, 50, 75, and 100% vol were prepared. Raman spectra were recorded using the portable system and a rectangular and a rounded colorless glass container, each with a 1.5 mm thickness, as sample holders. The intensity (height) of the main ethanol peak at 878 cm^−1^ was measured after background removal and plotted as a function of concentration (Figure 7). The intensities measured for the two sample holders were similar. Thus, the results were pooled and a general calibration equation was derived:

I_EtOH, 878cm−1_ = −132.5934 (±340.5896) + 305.8411 (±5.5618) C_EtOH_ (% vol) (1)

R^2^ = 0.998

### 3.7. Calibration Testing

#### 3.7.1. Repeatability

Intraday and Interday tests were performed using the standard ethanol–water solutions with concentrations of 25, 50, 75, and 100% vol in order to verify the repeatability of the results. All data are quoted in Table 3.

The repeatability of the results is considered satisfactory, being less than 5% in most cases for intraday analysis and less than 4% for the interday analysis.

#### 3.7.2. Robustness

- Bottle shape

The bottle shape did not affect the results. The calibration line was similar either for circular or rectangular glass containers, as is shown in Figure 7.

- Bottle color

Colored containers were found to reduce Raman intensity. In the case that a quantitative analysis of a spirit placed in a dark container is performed, a calibration line should be constructed using the same container as the spirit.

- Liquid (spirit, beverage) color

The effect of the color of the liquid is studied below (Section 3.9).

### 3.8. Limits of Detection and Quantitation

The limits of detection (LOD) and quantitation (LOQ) for ethanol in the spirits were calculated [28] and found to be 0.50% vol and 1.12% vol, respectively, which are much lower than the concentration of interest (4.5% vol, Table 1). A synthetically prepared mixture of 0.5% vol ethanol in water confirmed the calculated LOD (the ethanol peak was barely visible).

### 3.9. Method Application

#### 3.9.1. Colorless Spirits

The previously described methodology was applied for the calculation of the concentration of colorless spirits. The results and the relative error with respect to the nominal concentration (bottle label) are quoted in Table 4. There is good agreement between them. 

#### 3.9.2. Slightly Colored Spirits and Beverages

The calibration curve previously constructed (Equation (1)) was successfully extended to slightly colored spirits (whiskeys) (Table 4).

However, the same Equation (1) proved unsuitable for beers, though they were also slightly colored (light yellow). This is probably due to the presence of pressurized CO_2_. For the construction of an appropriate calibration curve, beers with different ethanol contents (Blue Island, 0% vol; Carib, 5% vol; and Desperados, 5.9% vol) were used as standard solutions. A linear regression provided the following equation:

I_EtOH, 883cm−1_ = 8.6852 (±74.6323) + 476.8756 (±16.7148) C_EtOH_ (% vol), R^2^ = 0.9975 (2)

Another beer (Somersby, 4.5% vol) was used to test the validity of the calibration Equation (2). The concentration, calculated using the equation, was 4.44% vol (relative error to the nominal value: 1.38%). It is worthy to note that beers from different firms were used at this stage of analysis.

#### 3.9.3. Dark-Colored Spirits

Comparing the spectra of liqueurs (dark-colored spirits) and whiskeys (light-colored spirits) in Figure 6, it can be seen that, although the concentrations are approximately the same, the intensities are not comparable. The darker the color of the spirit, the lower the intensity of ethanol peaks. This suggests that different calibration curves should be constructed and used for colored spirits. The standard addition method can be also used, but this implies the unsealing of the bottle, which is destructive and invasive for the product (not an in-line procedure).

### 3.10. Illicit Alcohols in Spirits (Methanol and Isopropanol)

The presence of illicit alcohols (methanol and isopropanol) in spirits was also investigated using the portable Raman setup and the “through the container” methodology.

**Figure 8:**
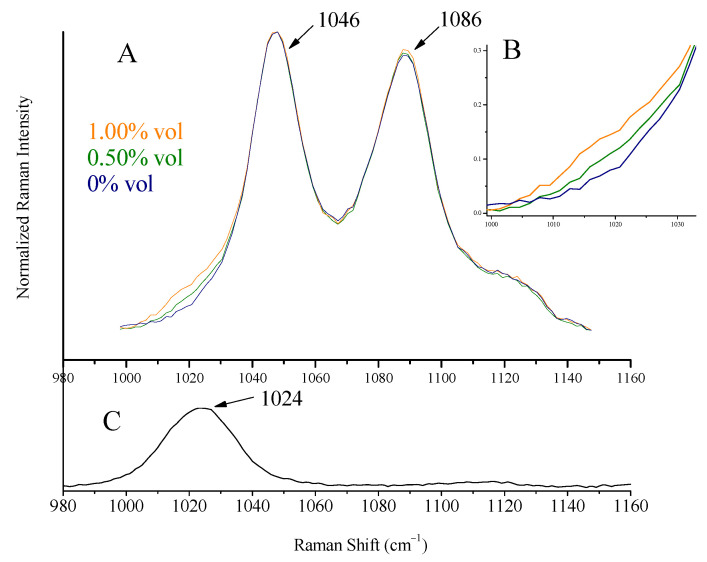
(**A**) Raman spectra of increasing concentrations of methanol (0–1.00% vol) added to Ballantine’s whiskey. The peak of methanol appears as a shoulder on the left of the ethanol peak at 1046 cm^−1^. The spectra quoted are normalized to the 1046 cm^−1^. (**B**) The magnified spectral area, 1000–1030 cm^−1^, of (**A**). Methanol presence can be verified for a content of 0.40% vol or higher. (**C**) Raman spectrum of methanol–water solution (1:1).

**Figure 9:**
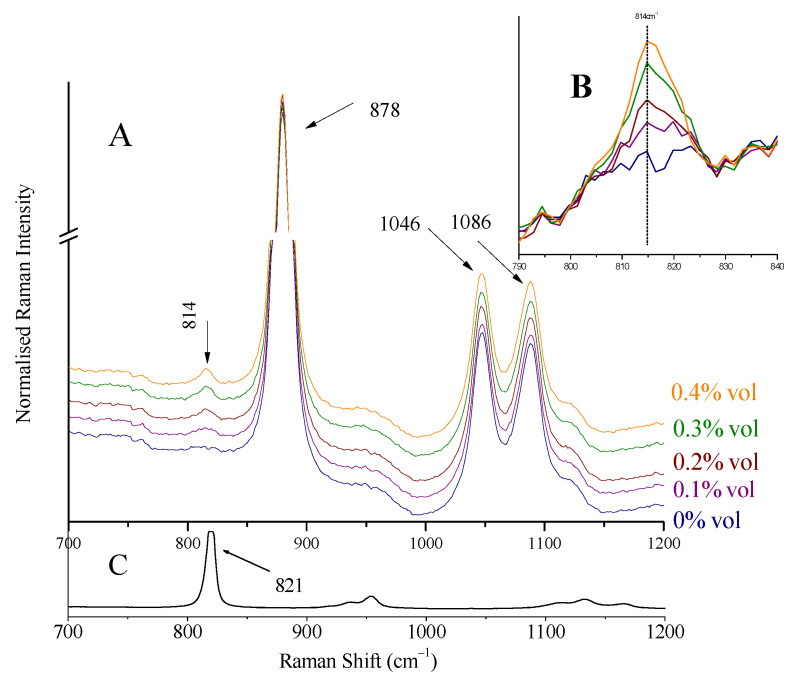
(**A**) Raman spectra of increasing concentrations of isopropanol (0–0.40% vol) added to Ballantine’s whiskey. The peak of isopropanol appears at 814 cm^−1^. The spectra quoted are normalized to the 878 cm^−1^ peak of ethanol. (**B**) Magnified spectral area 790–840 cm^−1^ of (**A**). The spectra are baselined. Isopropanol presence can be verified for content 0.10% vol or higher. (**C**) Raman spectrum of isopropanol.

The most intense peak of methanol at 1035 cm^−1^, attributed to C-O stretching vibration [29], is shifted to 1024 cm^−1^ in the spirit’s environment and appears as a shoulder on the left of ethanol peak at 1046 cm^−1^ (Figure 8). Isopropanol’s major peak at 821 cm^−1^, assigned to C-C stretching of C-C-O vibration [30,31], shifted to 814 cm^−1^ (Figure 9). These were the peaks used for detection and quantification of the alcohols. 

Solutions (two series) with precalculated concentrations of methanol (0.20, 0.40, 0.80, 1.00, and 1.50% vol) and isopropanol (0.10, 0.20, 0.30, and 0.40% vol) were prepared (separately for each alcohol) by the addition of the appropriate quantities of the alcohols to whiskey (Ballantine’s, 43% vol). Raman spectra were collected using the whiskey glass container as sample holder. Characteristic spectra are quoted in Figure 8 (for methanol) and Figure 9 (for isopropanol). The peaks of methanol at 1024 cm^−1^ (shoulder on the left side of the ethanol peak at 1046 cm^−1^) and isopropanol at 814 cm^−1^ can be seen. The spectra in the Figures are normalized as to the major peak, for clarity.

### 3.11. Measuring Illicit Alcohols Content

A calibration curve was constructed for each alcohol using the standard solutions prepared previously and their Raman spectra, pictured in Figure 8 and Figure 9. After appropriate baselining of the alcohols’ peaks (shoulder at 1024 cm^−1^ for methanol and peak at 814 cm^−1^ for isopropanol), their intensity ratios to ethanol (peaks at 1046 cm^−1^ and 878 cm^−1^) were sketched as a function of concentration (Equations (3) and (4)). The background signal, i.e., the respective ratios for the blank solution (pure whiskey), was subtracted. The calculation of these mathematical expressions was considered necessary as toxic alcohol content was (and is expected to be) low, and the signal from the ethanol is significant (see Figure 9B). 

The calibration curves (3 and 4) are quoted in the following. They exhibited good linearity, although the concentrations were lower than limit of quantitation. The spectra of minor alcohol additions were not used.

For methanol:

(I_1024cm−1_/I_1046cm−1_) − (I_blank, 1024cm−1_/I_blank, 1046cm−1_) = 
−0.00214 (±0.01292) + 0.07741 (±0.01210) C_MeOH_ (% vol) (3)

R^2^ = 0.95, for a concentration range 0.8–1.5% vol.

For isopropanol:

(I_814cm−1_/I_878cm−1_) − (I_blank, 814cm−1_/I_blank, 878cm−1_) =
−0.00004 (± 0.00206) + 0.04495 (± 0.00752) C_iPrOH_ (% vol) (4)

R^2^ = 0.92, for a concentration range 0.2–0.4% vol.

Intraday and interday analyses (Table 5 and Table 6) verified the repeatability of the results for both cases.

The detection limits calculated from the calibration curves were 0.39% vol for methanol and 0.16% vol for isopropanol, respectively. They were both verified from the spectra in Figure 8 and Figure 9. 

It should be mentioned that no methanol or isopropanol was detected in any of the commercially available spirits and liquors studied in the present work.

## 4. Discussion

The present work sought a method for the quick measurement of ethanol and illicit alcohols in beverages and spirits. This method will compensate for the disadvantages of the commonly used chromatographic techniques, which are time-consuming, require bottle removal from the production line and transfer to the lab, and sample preparation, which is different for ethanol in the case of the low-content alcohols (methanol and isopropanol). All these factors imply offline application, long analysis times, and delays in production and product availability.

The selected technique was Raman spectroscopy, which is known for its non-destructive character and fast analysis times. A modern, miniature, portable apparatus equipped with a fiber-optic accessory offers the possibility of direct on-site analysis, i.e., during production (in-line manner). 

The “through the container” method for the analysis of spirits and alcoholic beverages, developed and proposed in this work, provides a non-invasive and non-destructive character to the application. This means that there is no need to unseal the container and withdraw a sample for the analysis. The spirit/beverage is safe for consumption. 

Care should be taken regarding the focusing of the laser to maximize the Raman signal from the spirit/beverage and to suppress the signal from the glass (the probe should touch the glass wall in a vertical position, so the laser is focused in the liquid, through the glass and not on the glass or in front of it, outside the bottle). Any other position of laser spot (on the container wall, in front of the wall) will result in the maximization of the glass signal and a major suppression of the liquid signal.

The construction of calibration curves using standard solutions ensured the quick and cumulative character of the analysis. Thus, a large number of alcoholic beverages can be analyzed in a row under the same calibration curve. The time needed for one sample analysis can be as little as a few seconds. The portability of the device allows for application in any place, even in the production line. The non-necessity to transfer the beverage to the analysis lab enhances the speed of analysis.

The glass wall adds a broad peak to the spectrum. The glass signal can be suppressed to a large extent by following the instructions given for signal optimization (the fiber-optic probe should vertically touch the bottle wall so that the laser is focused in the liquid. For thick walls, the glass signal will not be eliminated, but it will not interfere with the alcohol signal to a significant degree, as ethanol peaks are well-separated from the broad glass peak). In any case, calibration should be constructed using the same bottle. Then the standard solutions will be recorded under the same experimental conditions as the spirits to be analyzed. 

The container materials include glass or plastic, though plastic is not very common. However, the color of the material may impose a restriction on the analysis. Dark-colored containers will absorb the incident radiation and the scattered signal from the sample will be suppressed to such a degree that analysis will be impossible to perform. In such cases, another portable system can be used. 

This method can practically be applied to any beverage or spirit. There can exist groups of spirits (e.g., colorless and slightly colored spirits) which follow the same calibration equation. For those which diverge, another, more appropriate calibration curve must be constructed to solve the problem (e.g., beer, due to pressurized CO_2_, and dark-colored spirits, due to their color). 

Three alcohols can be most often found in spirits: ethanol and, in some cases, methanol and/or isopropanol. The last two are very dangerous. An ethanol content in human blood of 5.07 μL/mL is considered lethal. The respective value for methanol is referred to be as low as 0.49 μL/mL [32], and it is 430 mg/lt for isopropanol [33]. 

The three alcohols, in case that they coexist in a spirit, can be analyzed simultaneously in the same spectrum without the need for a more complex sample preparation (e.g., a dilution to decrease ethanol content, and methanol/isopropanol direct measurement due to low content). Care should be taken, however, to apply the appropriate calibration equation for each alcohol; they do not follow the same equation as they are different molecules and exhibit a different Raman spectrum with different peaks. 

The level of alcohol content does not complicate the procedure. Alcohol constituents of a large concentration (ethanol) can be analyzed simultaneously in the same spectrum with the low-content (toxic) alcohols. 

The lower concentrations that were possible to measure (at the limit of detection) were 0.50% vol, 0.39% vol, and 0.16% vol for ethanol, methanol, and isopropanol, respectively. The ethanol content in spirits and beverages is usually well higher than 0.50% vol (e.g., 40% in spirits and 4–6% in beers). A measurable content of methanol of 0.39% vol is lower than the maximum tolerable level in spirits for an adult weighing 70 kg who consumes 100 mL of a spirit at 40% vol (14,000 mg methanol/L spirit, which is 1.4% vol) [34]. It is also acceptable against the general limit for naturally occurring methanol, which is 10 g/L ethanol, corresponding to 0.4% vol methanol in a 40% vol alcohol spirit [35]. 

The calibration curves presented here were validated to ensure the repeatability of results. Intraday and interday tests verified the method robustness. Validation was successful for aquatic solutions (ethanol in water) as standards as well as standards prepared in a spirit environment (the addition of methanol and isopropanol to whiskey).

## 5. Conclusions

In the present work, as was thoroughly analyzed in the previous sections, a method for the measurement of ethanol and illicit alcohols (methanol and isopropanol) in beverages and spirits was developed. The method relied on a miniature, portable Raman spectroscopy apparatus equipped with a fiber-optic accessory. A “through the container” setup was proposed.

Major advantages of this approach include the direct character of the analysis, i.e., in the production line (in-line analysis), and the non-destructive and especially the non-invasive application, which implies the non-necessity for the unsealing of the bottle to withdraw a sample to another lab at any stage of the analysis. The procedure is simple, quick, and non-strenuous.

The three alcohols, in the case that they coexist in a spirit, can be analyzed simultaneously in the same spectrum with no necessity of more complex sample preparation, i.e., dilution due to the large concentration of ethanol and an additional analysis cycle.

Calibration curves were constructed, checked, and validated with intraday and interday tests to ensure the repeatability of results. Detection limits for all alcohols were calculated and were found to be lower than the tolerable levels for human consumption.

This method can practically be applied to any beverage or spirit. Spirits may be grouped together (e.g., colorless and slightly colored spirits) to follow the same calibration equation. For those which diverge, another more appropriate calibration curve must be constructed.

On the other hand, care should be taken when focusing the laser to maximize the Raman signal from the spirit/beverage and to suppress the signal from the container. However, the scattering from the glass, even in cases that it cannot be eliminated due to increased glass thickness, will not interfere with the alcohol to such a significant degree that analysis is considered insecure.

The calibration curve should be constructed using the same container and experimental conditions as the spirits to be analyzed. It should be also in the analyst’s mind that not all the spirits/beverages may be analyzed using the same calibration. For some of them (e.g., beers) another calibration should be constructed and followed.

A restriction is also posed by the color of the liquid. Dark-colored spirits will exhibit a suppressed signal due to laser absorption.

## Figures and Tables

**Table 1:** Drinks (brand name, type, volume of the container) used for the study. The alcohol content (strength), color, and as information about the container are quoted.

Brand Name (Type of Drink, Alcoholic Strength, Volume)	Characteristics of the Drink (^1,2^)	Characteristics of the Bottle (^3,4,5^)
Blue island (Beer, 0%), 330 mL	light yellow	
Blue island (Beer, 4.5%), 330 mL	light yellow	
Carib (Beer, 5%), 330 mL	light yellow	
Desperados (Beer, 5.9%), 250 mL	light yellow	
Somersby (Beer, 4.5%), 330 mL	light yellow	
Martell (Cognac, 40%), 50 mL	dark brown, not tranparent	
Beefeater (Gin, 47%), 50 mL		plastic
Ahududu (Liqueur, unknown), 50 mL	dark red, not transparent	
Banane (Liqueur, unknown), 50 mL	yellow	
Belle epoque (Liqueur, unknown), 50 mL	orange, not transparent	
Brandy (Liqueur, unknown), 50 mL	light yellow	
Drambuie (Liqueur, 40%), 50 mL	yellow	plastic, brown, not transparent
Fraise (Liqueur, unknown), 50 mL	red, not transparent	
Limoncello (Liqueur, 32%), 50 mL	yellow, not transparent	
Marascino (Liqueur, unknown), 50 mL	light yellow	
Sabra (Liqueur, 30%), 50 mL	burgundy, not transparent	
Tikelli (Ouzo, 40%), 50 mL		
Varvagianni (Ouzo, 42%), 50 mL		
Haraki (Raki, 40%), 50 mL		
Agioritiko (Tripouro, 38%), 50 mL		
Amorgos (Tsipouro, 20%), 50 mL	dark brown, not transparent	
Babajim (Tsipouro, 40%), 50 mL		
Stolichnaya (Vodka, 40%), 50 mL		
Ballantines (Whiskey, 43%), 50 mL	light yellow	
Jack Daniel’s (Whiskey, 43%), 50 mL	light yellow	
J & B (Whiskey, 40%), 50 mL	light yellow	plastic, green, not transparent
Vat 69 (Whiskey, 40%), 700 mL	yellow	green, not transparent
Kourtaki (Wine, 11.5%), 500 mL	light yellow	

^1^ All drinks are colorless, unless otherwise stated. ^2^ All drinks are transparent, unless otherwise stated. ^3^ All bottles are made of glass, unless otherwise stated. ^4^ All bottles are colorless, unless otherwise stated. ^5^ All bottles are transparent, unless otherwise stated.

**Table 2:** Peak maximum and assignment for pure ethanol and ethanol in whiskey.

Ethanol Peak Maximum (cm^−1^)	Whiskey Peak Maximum (cm^−1^)	Band Assignment
433	433	C-C-O bending
883	878	C-C-O stretching
1053	1046	C-C-O stretching
1097	1086	C-O stretching, CH_3_ rocking
1277	1276	C-H torsion and rotation
1454	1454	CH_3_ and CH_2_ bending
1486	1483	CH_3_ bending

**Table 3:** Interday (A) and intraday (B) repeatability tests for ethanol–water standard solutions.

**A. Interday**			
**C_EtOH_** **(% vol)**		**Calculated C_EtOH_ ± SD** **(% vol)**	**Rel. Error (%)**
25	1st day	26.57 ± 1.99	6.29
	2nd day	24.94 ± 0.80	0.24
	3rd day	26.01 ± 1.76	4.05
50	1st day	49.67 ± 1.85	0.65
	2nd day	50.51 ± 1.86	1.02
	3rd day	47.84 ± 2.11	4.33
75	1st day	70.37 ± 2.16	6.18
	2nd day	74.95 ± 1.70	0.06
	3rd day	74.89 ± 4.09	0.15
100	1st day	100.06 ± 0.29	0.06
	2nd day	99.68 ± 2.91	0.32
	3rd day	104.43 ± 5.16	4.43
**B. Intraday**			
**C_EtOH_** **(% vol)**	**Calculated C_EtOH_ ± SD** **(% vol)**		**Rel. Error (%)**
25	25.91 ± 1.67		3.62
50	49.34 ± 2.14		1.32
75	73.40 ± 3.45		2.13
100	101.39 ± 3.88		1.39

**Table 4:** Ethanol concentration in spirits calculated using the portable Raman setup and the “through the container” method. Deviation to the label value (relative error) is also quoted (*).

Drink Analyzed	Color	Calculated Concentration (%)	Relative Error (%)
Ouzo, Varvagiani (42% vol)	colorless	42.17 ± 1.44	0.40
Whiskey, Ballantines (43% vol)	light yellow	43.80 ± 1.30	1.86
Whiskey. Jack Daniels (43% vol)	light yellow	42.89 ± 1.80	0.24
Vodka, Stolochnaya (40% vol)	colorless	39.51 ± 2.37	1.22

(*) The calculated concentration is given as the mean ± standard deviation. The relative error is calculated as the absolute value of [(calculated concentration − label concentration)/label concentration] × 100%.

**Table 5:** Interday and intraday repeatability tests for the methanol–whiskey standard solutions.

**A. Interday**			
**C_MeOH_** **(% vol)**		**Calculated C_MeOH_ ± SD** **(% vol)**	**Rel. Error (%)**
0.8	1st day	0.835 ± 0.073	4.4
	2nd day	0.812 ± 0.049	1.5
	3rd day	0.779 ± 0.095	2.6
1.0	1st day	0.968 ± 0.089	3.2
	2nd day	1.083 ± 0.086	8.3
	3rd day	0.914 ± 0.063	8.6
1.5	1st day	1.532 ± 0.091	2.1
	2nd day	1.491 ± 0.085	0.6
	3rd day	1.540 ± 0.097	2.7
**B. Intraday**			
**C_MeOH_** **(% vol)**	**Calculated C_MeOH_ ± SD** **(% vol)**		**Rel. Error (%)**
0.8	0.814 ± 0.035		1.73
1.0	0.870 ± 0.084		12.97
1.5	1.512 ± 0.052		0.79

**Table 6:** Interday and intraday repeatability tests for the isopropanol–whiskey standard solutions.

**A. Interday**			
**C_iPrOH_** **(% vol)**		**Calculated C_iPrOH_ ± SD** **(% vol)**	**Rel. Error (%)**
0.2	1st day	0.167 ± 0.045	16.5
	2nd day	0.185 ± 0.030	7.5
	3rd day	0.201 ± 0.024	0.5
0.3	1st day	0.315 ± 0.016	5.0
	2nd day	0.291 ± 0.018	3.0
	3rd day	0.331 ± 0.020	10.3
0.4	1st day	0.402 ± 0.015	0.5
	2nd day	0.397 ± 0.021	0.8
	3rd day	0.405 ± 0.019	1.3
**B. Intraday**			
**C_iPrOH_** **(% vol)**	**Calculated C_iPrOH_ ± SD** **(% vol)**		**Rel. Error (%)**
0.2	0.158 ± 0.065		20.85
0.3	0.326 ± 0.012		8.77
0.4	0.396 ± 0.025		0.96

## Data Availability

Not applicable.

## Supplementary Materials

The following supporting information can be downloaded at: https://www.mdpi.com/article/10.3390/bios13010135/s1, Figure S1: Raman spectra of colorless spirits; Figure S2: Raman spectra of yellow-color spirits; Figure S3: Raman spectra of red-color spirits.

## References

[1] Nordon A., Mills A., Burn R.T., Cusick F.M., Littlejohn D. (2005). Comparison of non-invasive NIR and Raman spectrometries for determination of alcohol content of spirits. Anal. Chim. Acta.

[2] Lachenmeier D.W., Godelmann R., Steiner M., Ansay B., Weigel J., Krieg G. (2010). Rapid and mobile determination of alcoholic strength in wine, beer and spirits using a flow-through infrared sensor. Chem. Cent. J..

[3] Wang M.-L., Wang J.-T., Choong Y.-M. (2004). A rapid and accurate method for detrmination of methanol in alcoholic beverage by direct injection capillary gas chromatography. J. Food Compos. Anal..

[4] Calull M., Marce R.M., Borull F. (1992). Determination of carboxylic acids, sugars, glycerol and ethanol in wine and grape must by ion-exchange high-performance liquid with refractive index detection. J. Chromatogr..

[5] MacKenzie W.M., Aylott R.I. (2004). Analytical strategies to confirm Scotch whiskey authenticity. Part II: Mobile brand authentication. Analyst.

[6] Iñón F.A., Llario R., Garrigues S., de La Guardia M. (2005). Development of a PLS based method for determination of the quality of beers by use of NIR: Spectral ranges and sample-introduction considerations. Anal. Bioanal. Chem..

[7] Cavinato A.G., Mayes D.M., Ge Z., Callis J. (1990). B Noninvasive method for monitoring ethanol in fermentation processes using fiber-optic near-infrared spectroscopy. Anal. Chem..

[8] Tipparat P., Lapanantnoppakhun S., Jakmunee J., Grudpan K. (2001). Determination of ethanol in liquor by near-infrared spectrophotometry with flow injection. Talanta.

[9] Sato-Berrú R.Y., Medina-Valtierra J., Medina-Gutiérrez C., Frausto-Reyes C. (2004). Quantitative NIR Raman analysis in liquid mixtures. Spectrochim. Acta Part A Mol. Biomol. Spectrosc..

[10] Yang Y.R., Ren Y.F., Dong G.M., Yang R.J., Liu H.X., Du Y.H., Zhang W.Y. (2016). Determination of Methanol in Alcoholic Beverages by Two-Dimensional Near-Infrared Correlation Spectroscopy. Anal. Lett..

[11] Debebe A., Anberbir A., Redi-Abshiro M., Chandravanshi B.S., Asfaw A., Asfaw N., Retta N. (2017). Alcohol determination in distilled alcoholic beverages by liquid phase Fourier transform mid-infrared and near-infrared spectrophotometers. Food Anal. Methods.

[12] Pontes M.J.C., Santos S.R.B., Araujo M.C.U., Almeida L.F., Lima R.A.C., Gaiao E.N., Souto U.T.C.P. (2006). Classification of distilled alcoholic beverages and verification of adulteration by near infrared spectrometry. Food Res. Int..

[13] Klein O., Roth A., Dornuf F., Schöller O., Mäntele W. (2012). The Good Vibrations of Beer. The Use of Infrared and UV/Vis Spectroscopy and Chemometry for the Quantitative Analysis of Beverages. Z. Für Nat. B.

[14] Fearn T., Smith D.B., Starr C., Halsey S.A. (1986). Application of near infrared spectroscopy in the food industry. Anal. Proc..

[15] Engelhard S., Löhmannsröben H.-G., Schael F. (2004). Quantifying Ethanol Content of Beer Using Interpretive Near-Infrared Spectroscopy. Appl. Spectrosc..

[16] Gallignani M., Garrigues S., de la Guardia M. (1993). Direct determination of ethanol in all types of alcoholic beverages by near-infrared derivative spectrometry. Analyst.

[17] Mendes L.S., Oliveira F.C.C., Suarez P.A.Z., Rubin J.C. (2003). Determination of ethanol in fuel ethanol and beverages by Fourier transform (FT)-near infrared and FT-Ramn spectrometries. Anal. Chim. Acta.

[18] Barboza F.D., Poppi R.J. (2003). Determination of alcohol content in beverages using short-wave near-infrared spectroscopy and temperature correction by transfer calibration procedures. Anal. Bioanal. Chem..

[19] Wang Q., Li Z., Ma Z., Si G. (2015). Quantitative Analysis of Multiple Components in Wine Fermentation using Raman Spectroscopy. Adv. J. Food Sci. Technol..

[20] Boyaci I.H., Genis H.E., Guven B., Tamer U., Alper N. (2012). A novel method for quantification of ethanol and methanol in distilled alcoholic beverages using Raman spectroscopy. J. Raman Spectrosc..

[21] Ashok P.C., Praveen B.B., Dholakia K. (2013). Optofluidic Raman sensor for simultaneous detection of the toxicity and quality of alcoholic beverages. J. Raman Spectrosc..

[22] De Goes R.E., Fabris L.V.M., Muller M., Fabris J.L. (2016). Light-Assisted Detection of Methanol in Contaminated Spirits. J. Light. Technol..

[23] Frausto-Reyes C., Medina-Gutiérrez C., Sato-Berrú R., Sahagún L.R. (2005). Qualitative study of ethanol content in tequilas by Raman spectroscopy and principal component analysis. Spectrochim. Acta Part A Mol. Biomol. Spectrosc..

[24] Teixeira Dos Santos C.A., Páscoa R.N.M.J., Porto P.A.L.S., Cerdeira A.L., González-Sáiz J.M., Pizarro C., Lopes J.A. (2018). Raman spectroscopy for wine analyses: A comparison with near and mid infrared spectroscopy. Talanta.

[25] Zeren C., Acikgoz G., Kahraman S. (2017). Using Raman spectroscopy for determination methanol quantity in illegal alcoholic beverages. Spectrosc. Spectr. Anal..

[26] Abe N., Ito M. (1978). Effects of hydrogen bonding on the Raman intensities of methanol, ethanol and water. J. Raman Spectrosc..

[27] Burikov S., Dolenko T., Patsaeva S., Starokurov Y., Yuzhakov V. (2010). Raman and IR spectroscopy research on hydrogen bonding in water-ethanol systems. Mol. Phys..

[28] Miller J.N., Miller J.C. (2005). Calibration methods: Regression and Correlation. Statistics and Chemometrics for Analytical Chemistry.

[29] Matsuura H., Yamamoto M., Murata H. (1980). Raman spectra and normal vibrations of methylene glycol and its perdeuterated analogue. Spectrochim. Acta Part A Mol. Spectrosc..

[30] Jin Z., Chu Q., Xu W., Cai H., Ji W., Wang G., Lin B., Zhang X. (2018). All-Fiber Raman Biosensor by Combining Reflection and Transmission Mode. IEEE Photonics Technol. Lett..

[31] Yang B., Cao X., Wang S., Sun C. (2020). Exploring molecular association of isopropanol-water binary solution by Raman spectroscopy. Optik.

[32] McCoy H.G., Cipolle R.J., Ehlers S.M., Sawchuk R.J., Zaske D.E. (1979). Severe methanol poisoning: Application of a pharmacokinetic model for ethanol therapy and hemodialysis. Am. J. Med..

[33] Hydara Y.E., Zilg B. (2020). Postmortem diagnosis of ketoacidosis: Levels of beta-hydroxybutyrate, acetone and isopropanol in different causes of death. Forensic Sci. Int..

[34] Lachenmeier D.W., Haupt S., Schulz K. (2008). Defining maximum levels of higher alcohols in alcoholic beverages and surrogate alcohol products. Regul. Toxicol. Pharmacol..

[35] Botelho G., Anjos O., Estevinho L.M., Caldeira I. (2020). Methanol in grape derived, fruit and honey spirits: A critical review on source, quality control and legal limits. Processes.

