# Supplementary material for: Non-Destructive and Non-Invasive Measurement of Ethanol and Toxic Alcohol Strengths in Beverages and Spirits Using Portable Raman Spectroscopy
Source: Biosensors (Basel). 2023 Jan 13;13(1):135. doi: 10.3390/bios13010135 (PMC9856296; doi:10.3390/bios13010135)
Supplement: Supplementary file 1 [file biosensors-13-00135-s001.zip › biosensors-2136415-supplementary.pdf]

Supplementary Data

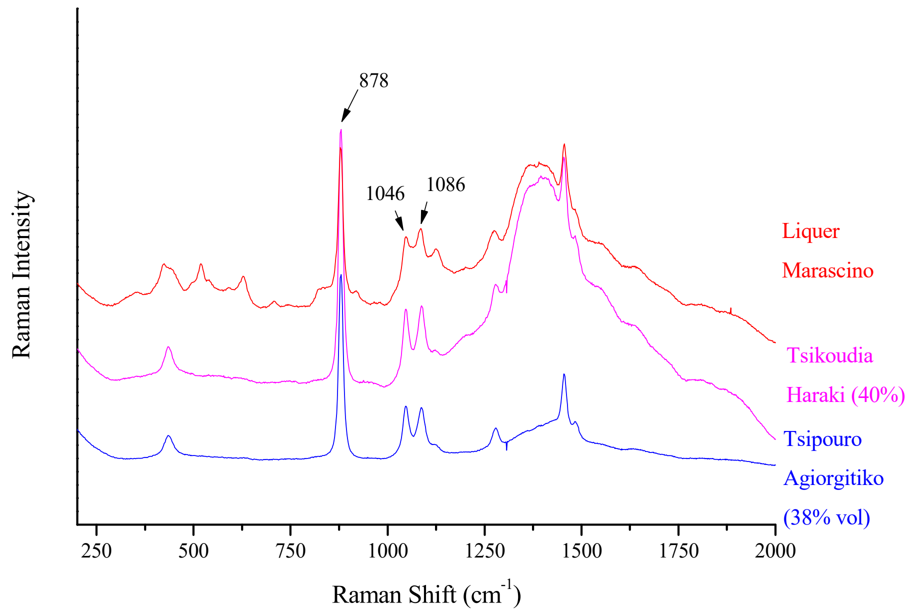

Figure S1. Raman spectra of colorless spirits.

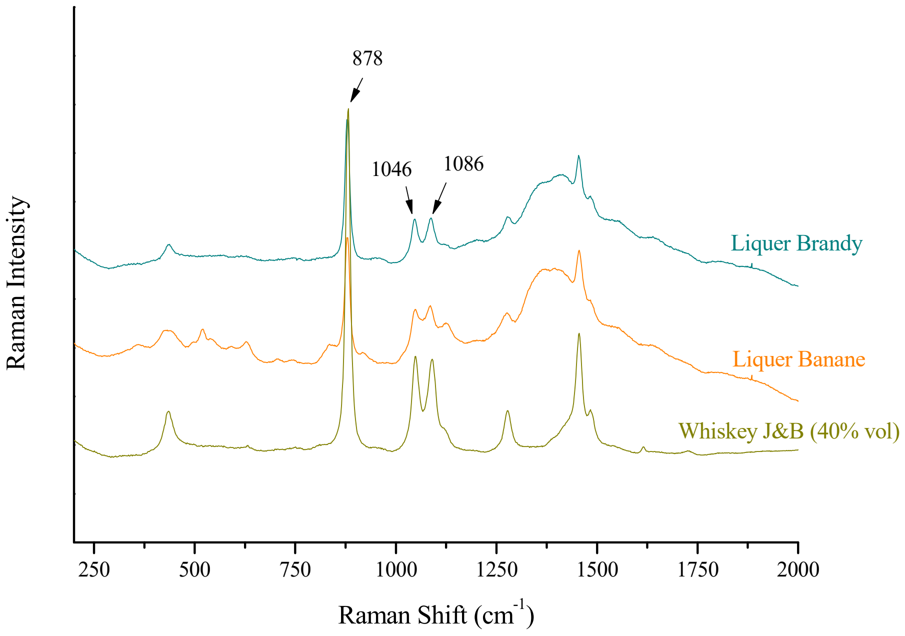

Figure S2. Raman spectra of yellow-color spirits.

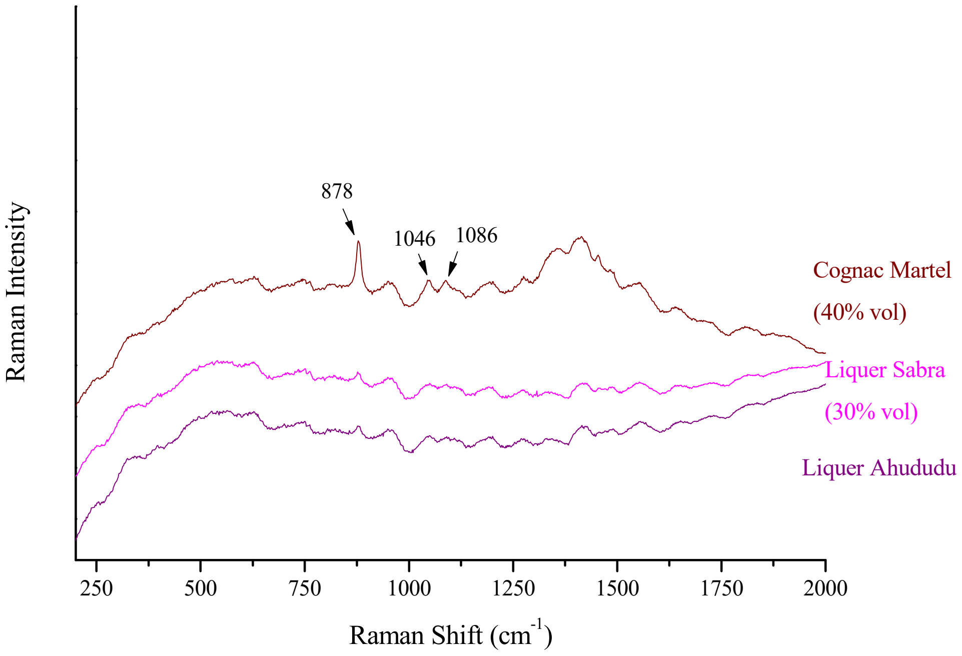

Figure S3. Raman spectra of red-color spirits.
